# The mediating role of complex posttraumatic stress and borderline pattern symptoms on the association between sexual abuse and suicide risk

**DOI:** 10.1186/s40479-022-00183-z

**Published:** 2022-04-12

**Authors:** Odeta Gelezelyte, Monika Kvedaraite, Agniete Kairyte, Neil P. Roberts, Jonathan I. Bisson, Evaldas Kazlauskas

**Affiliations:** 1 Center for Psychotraumatology, Institute of Psychology, Vilnius University, M. K. Ciurlionio str. 29, Vilnius, Lithuania; 2 Division of Psychological Medicine and Clinical Neurosciences, Cardiff University School of Medicine, Cardiff, UK; 3 Psychology and Psychological Therapies Directorate, Cardiff & Vale University Health Board, Cardiff, UK

**Keywords:** complex posttraumatic stress disorder, borderline, suicide risk, suicidal behavior, sexual abuse

## Abstract

**Background:**

The 11th revision of the International Classification of Diseases (ICD-11) includes a new diagnosis of complex posttraumatic stress disorder (CPTSD). There has been very little research investigating associations between CPTSD symptoms and suicide risk following sexual abuse. This and questions concerning similarities and differences between CPTSD and borderline personality disorder (BPD), led to the current study that aimed to explore indirect associations between sexual abuse and suicide risk through the symptoms of CPTSD and borderline traits.

**Methods:**

The study sample comprised 103 adults with a history of traumatic experiences (*M*_*age*_ = 32.64, *SD*_*age*_ = 9.36; 83.5% female). In total, 26.3% of the participants reported experiencing sexual abuse during their lifetime. The clinician-administered International Trauma Interview (ITI) was used for the assessment of ICD-11 CPTSD symptoms. Self-report measures were used for the evaluation of borderline pattern (BP) symptoms and suicide risk. Mediation analyses were performed to evaluate the mediating effects of CPTSD and BP symptoms for the association between sexual trauma and suicide risk.

**Results:**

In a parallel mediation model, CPTSD and BP symptoms mediated the association between sexual abuse and suicide risk, following adjustment for the covariates of age, gender, and whether the traumatic experience occurred in childhood or adulthood. Around 73% of participants who met diagnostic criteria for CPTSD reported previous suicide attempt(s).

**Conclusions:**

Suicide risk assessment and intervention should be an important part of the management of victims of sexual abuse with CPTSD and BP symptoms.

## Background

The 11th revision of the International Classification of Diseases (ICD-11) contains significant changes in the diagnostics of posttraumatic stress. In addition to posttraumatic stress disorder (PTSD), a new sibling disorder of complex posttraumatic stress disorder (CPTSD) has been included [1]. ICD-11 PTSD encompasses three symptom clusters: re-experiencing, avoidance, and persistent perception of the heightened current threat. CPTSD is diagnosed when, in addition to all PTSD symptoms, a person experiences clinically significant levels of disturbances in self-organization (DSO), namely difficulties with affect regulation, negative self-concept, and disturbed relationships [1]. Both PTSD and CPTSD may develop following exposure to an event or series of traumatic events and cause significant functional impairment. A person can be diagnosed with either PTSD or CPTSD, but not both.

There was also a significant change in the diagnostics of personality disorders in the ICD-11 [2]. Instead of categorical description, a personality disorder is conceptualized along a general dimension of severity (mild, moderate, severe) and a five-domain dimensional trait model [1]. However, the ICD-11 revision also includes a specification of borderline pattern (BP) specifier, retaining close consistency with the DSM-5 criteria for borderline personality disorder (BPD) [3].

The proposal of the new CPTSD diagnosis raised questions about its overlap with borderline personality disorder [4]. Despite the debate, differences between CPTSD and BPD have been noted descriptively as well as demonstrated empirically [5]. Emotional lability and dyscontrol, unstable, fragmented sense of self, and unstable relationships are characteristic of BPD. Whereas, difficulties in self-calming or/and emotional numbing when faced with stressors, stable, persistent sense of worthlessness, and stable avoidance and detachment from relationships are specific to CPTSD [5]. The results of a study using network analysis revealed that CPTSD represented related symptoms, but BPD symptom network was rather sparse and weakly connected to CPTSD symptoms [6]. Another research using latent class analysis approach has demonstrated that CPTSD symptoms are distinguishable from BPD symptoms [7]. However, other analyses in various samples have also shown some overlap between symptom profiles of CPTSD and BPD [8, 9]. Here it is important to note that most of the studies following the official diagnostic guidelines of the ICD-11 CPTSD have used self-report measures to assess the symptoms. Participants might find it challenging to evaluate their DSO symptoms accurately, based on simply worded self-report items, and differentiate them from BPD symptoms, as understanding such nuances requires clinical judgment. More in-depth clinical assessment could provide more accurate results, and the overlap between the CPTSD and BPD might actually not be that high.

Another proposed difference between CPTSD and BPD is related to suicidal behavior. Previous studies demonstrated increased odds of self-harm, suicide ideation, and attempts in BPD patients [10]. It is claimed that in CPTSD, suicide risk is lower, and self-harm behavior is less frequent [11]. Some research showed that suicidal and self-injurious behaviors were central to BPD but not CPTSD [12]. In another study, after conducting latent class analysis, it was found that almost half of the individuals in the BPD class endorsed self-harm or suicidal behaviors, while this percentage was much lower in the PTSD and CPTSD groups [7]. Although there is evidence that PTSD is a risk factor for suicide ideation, suicide attempts, and deaths by suicide [13–15], there is a significant lack of studies exploring associations between a new diagnostic category of complex posttraumatic stress and suicide risk.

Sexual abuse is a risk factor for the development of both complex PTSD and borderline personality disorder. Meta-analyses showed that sexual abuse was associated with five times greater odds of receiving a BPD diagnosis among young people [10]. Childhood sexual abuse specifically increases the risk for the development of BPD [16]. In one study, CPTSD diagnosis, in comparison to PTSD diagnosis, was associated with experiencing adult sexual assault and other unwanted sexual experiences [17]. These findings were in line with another study, which reported 8% PTSD and 43% CPTSD prevalence among treatment-seeking adult victims of childhood sexual abuse [18]. Another study also found significant associations between childhood sexual abuse and increased risk for heightened PTSD, DSO, and BPD symptoms [8].

Moreover, there is evidence of a relationship between sexual abuse and suicide risk among people with BPD. For example, in one study, it was observed that people with BPD who experienced prolonged childhood sexual abuse attempted suicide more often and demonstrated higher severity of non-suicidal self-injury than people with BPD with no prolonged child sexual abuse [19]. In addition, suicidal BPD patients report experiencing significantly more events related to sexual abuse than non-suicidal patients with BPD [20].

In summary, there is a growing body of evidence on the associations between sexual trauma and symptoms of borderline pattern (BP) and CPTSD, as well as suicide risk. Although theoretical understanding alongside a few studies provide us with some information on suicide risk being higher among individuals with BPD, in comparison to those with CPTSD, the empirical evidence is still very limited. As CPTSD is a new diagnosis, there is still very little research on the links between CPTSD and suicidality. Furthermore, to our knowledge, no studies have investigated these links using a clinician-administered interview for diagnosing ICD-11 CPTSD so far. For the development of specific effective interventions, it is important to understand better the mechanisms that lead to higher suicide risk after experiencing sexual abuse. Therefore, the aim of the current study was to explore the mediating role of CPTSD and BP symptoms for the association between sexual abuse and suicide risk.

## Methods

### Participants and procedures

Participants were invited to take part in the study via social communication platforms and mental healthcare professionals across all regions of Lithuania. Inclusion criteria for this study were: (1) ≥18-years-old, (2) experienced at least one traumatic event during lifetime, (3) ≥3 months passed since the last experienced traumatic event, and (4) being able to communicate effectively in Lithuanian language. Data collection was divided into two parts: (1) filling in the self-report measures in a survey and (2) participating in a diagnostic interview with a clinical psychologist or supervised Master’s in Clinical Psychology student. All interviewers were professionally trained in how to administer and evaluate the International Trauma Interview (ITI). Data were collected from October 2020 to June 2021. Due to the COVID-19 (severe acute respiratory syndrome coronavirus 2, SARS-CoV-2) pandemic restrictions, the survey was taken using a secure online survey platform, and interviews were conducted via a videoconferencing platform. A more detailed description of the study procedure was reported previously [21].

The study sample comprised 103 adults, with age range from 18 to 54 years (*M*_*age*_ = 32.64, *SD*_*age*_ = 9.36), 83.5% were female. Most participants were Lithuanian (91.3%) and living in an urban area (94.2%). More than half (60.2%) of the sample had a university degree, 17.5% had a non-university higher education degree, and 19.4% had graduated from secondary education. Half of the participants (49.5%) were employed, 14.6% were studying, 15.5% were working and studying simultaneously, and one-fifth of participants (20.4%) were neither working nor studying.

### Measures

#### Posttraumatic and Complex Posttraumatic Stress Symptoms

The International Trauma Interview (ITI) [22] is a semi-structured diagnostic interview based on the ICD-11 PTSD and CPTSD symptom criteria. The ITI is comprised of a description of the index traumatic event(s) and PTSD and DSO symptom assessment sections.

The PTSD symptom assessment section contains the evaluation of the frequency and intensity of the following symptoms: (1) re-experiencing, (2) avoidance, and (3) sense of heightened current threat. Two items have to be evaluated in-depth for each symptom cluster. The severity of each symptom is rated by the interviewer on a five-point scale from absent (= 0) to extreme (= 4).

The DSO symptom assessment section is comprised of the evaluation of (1) affective dysregulation (hyper- or hypoactivation), (2) negative self-concept, and (3) disturbances in relationships. DSO symptoms have to be considered to be trauma related to contribute to diagnosis. The severity of each symptom is evaluated on a five-point scale from not at all (= 0) to extremely (= 4). The PTSD and DSO sections are followed by questions about functional impairment in the persons’ social life, work, or any other important area in life.

A symptom is confirmed as clinically significant if at least one of the two items measuring the symptom is evaluated as ≥2. PTSD can be diagnosed if at least one symptom in each symptom cluster and functional impairment related to the PTSD symptoms are evaluated as ≥2. CPTSD is confirmed if all PTSD diagnostic criteria are met, and at least one symptom in each DSO symptom cluster and functional impairment related to the DSO symptoms are evaluated as ≥2. The ITI requires trauma related DSO symptoms to have been present for at least three months for the diagnosis of the CPTSD. If the index trauma was recent but the individual has pre-existing trauma related DSO symptoms this would qualify.

In the current study, the intensity of the symptoms was evaluated by summing up the scores of each item from the PTSD and DSO symptom clusters. Scores for PTSD and DSO subscales may range from 0 to 24, giving a possible total ITI score range from 0 to 48. The psychometric properties of the Lithuanian version of ITI have previously been found to be robust [21]. In the current analysis, the Cronbach’s alpha coefficients of overall ITI (*α* = .87), as well as of separate PTSD (*α* = .81) and DSO (*α* = .84) sections, were good.

#### Symptoms of Borderline Pattern

The Borderline Pattern Scale (BPS) [3] was used to measure borderline personality pattern based on its description in the ICD-11. The BPS comprises four subscales, each with three items. The scale measures affective instability, maladaptive self-functioning, maladaptive interpersonal functioning, and maladaptive regulation strategies. Participants are asked to rate each item on a five-point scale from ‘strongly disagree’ (= 1) to ‘strongly agree’ (= 5). The score of the BPS is calculated by summing all the scores of the scale (ranging from 12 to 60), with a higher score indicating more severe symptoms. The Cronbach’s alpha coefficient of the total BPS in this sample was good (*α* = .82).

#### Suicide Risk

The Suicidal Behaviors Questionnaire-Revised (SBQ-R) [23] is a brief self-report measure used to evaluate four dimensions of suicidality. The first dimension is lifetime suicide ideation, and suicide attempts evaluated using a four-point scale, from ‘never’ (= 1) to ‘I have attempted to kill myself’ (= 4). The second dimension is the frequency of suicide ideation evaluated using a five-point scale from ‘never’ (= 1) to ‘very often’ (= 5). The third dimension is the threat of suicidal behavior evaluated by how often a person communicated about it to other people, using a three-point scale, from ‘no’ (= 1) to ‘yes, more than once’ (= 3). The last dimension includes the likelihood of suicidal behavior in the future, evaluated on a seven-point scale from ‘never’ (= 0) to ‘very likely’ (= 6). The final score of the SBQ-R is calculated by summing all items (ranging from 3 to 18). A higher score indicates more severe suicide risk. SBQ-R was used previously in studies in Lithuania [24]. The Cronbach’s alpha coefficient of the total SBQ-R scale in the current study was good (*α* = .81).

### Data Analysis

An independent-samples t-test, chi-square test for independence (post hoc testing was carried out after choosing the Bonferroni-corrected p-value: .05/3=.017), and bivariate correlations were conducted for the descriptive analyses. Next, we performed mediation analyses [25] with PROCESS macro v4.0 [26] in SPSS v 26. In the current study, to analyze to what extent each variable mediates the effect between sexual trauma (no experience of sexual abuse=0, experience of sexual abuse=1) and suicide risk (total score), conditional on the presence of the remaining mediators, all variables of interest were simultaneously included in a single parallel mediation model. In the parallel multiple mediator model the mediators were allowed to correlate but not causally influence another mediator in the model [27]. We firstly tested a parallel mediation model with the total scores of complex posttraumatic stress and borderline pattern symptoms included as mediators. Then, in the second model, the mediating role of the total scores of posttraumatic stress, disturbances in self-organization, and borderline pattern symptoms were tested. For the investigation of indirect effects, we selected the percentile method for bootstrapping with 10,000 bootstrap samples and 95% confidence interval [25, 27]. Age, gender (male=0, female=1), and trauma exposure in adulthood (=0) vs. childhood (=1) were included as covariates in the parallel mediation models. We obtained standardized coefficients for all continuous variables. Following the recommendations of Hayes [27], partially standardized regression coefficients were obtained for dichotomous predictors. As the variable of sexual trauma is dichotomous, the total, direct, and indirect effects are also presented in a partially standardized form.

## Results

### Descriptive statistics

Participants reported various index traumatic event(s) during the interview. The most often reported traumatic experience was physical abuse in childhood (20.4%; *N*=21). Participants also experienced sexual abuse in childhood (11.7%; *N*=12), unwanted sexual experiences in childhood (12.6%; *N*=13), sexual abuse in adulthood (14.6%; *N*=15), sudden violent death of a close person (14.6%; *N*=15), physical assault (4.9%; *N*=5); traffic accident (5.8%; *N*=6), and other traumatic experiences (15.4%; *N*=16). In total, 26.3% of the participants reported experiencing sexual abuse during their lifetime.

Means, standard deviations, and correlations of the study variables are presented in Table 1. The severity of suicide risk was significantly correlated with CPTSD and BP symptoms. Participants with the experience of sexual trauma had higher levels of suicide risk than the remaining sample (*M*=10.6, *SD*=3.79; *M*=8.32, *SD*=4.38; *t*(101)=-2.36, *p*=.020). Among participants with experience of sexual abuse, 63.0% (*N*=17) reported previous suicide attempts. The proportion was almost twice as high (χ^2^ (1, *N*=103) = 7.46, *p*=.006) as in the remaining sample, where 32.9% (*N*=25) participants reported attempting suicide. Participants with sexual trauma were also more likely to meet the requirements for the diagnosis of PTSD (40.7% (*N*=11) vs. 14.5% (*N*=11); χ^2^ (1, *N*=103) = 8.18, *p*=.004) as well as CPTSD (33.3% (*N*=9) vs. 13.2% (*N*=10); χ^2^ (1, *N*=103) = 5.39, *p*=.020) than the remaining sample. A higher percentage of participants with CPTSD reported a history of suicide attempts (no PTSD and no CPTSD: 27.4% (*N*=17), PTSD: 47.4% (*N*=9), CPTSD: 72.7% (*N*=16); χ^2^ (2, *N*=103) = 14.22, *p*=.001). The proportions of suicide attempts between the groups of no diagnosis and CPTSD diagnosis differed significantly (χ^2^ (1, *N*=84) = 13.98, *p*<.001).

**Table 1:** Means, standard deviations, and correlations among study variables

Variable	*M (SD)*	1	2	3	4
1. SBQ-R	8.9 (4.33)				
2. PTSD symptoms	6.53 (4.47)	.42***			
3. DSO symptoms	6.69 (5.25)	.37***	.55***		
4. CPTSD symptoms	13.22 (8.57)	.45***	.86***	.90***	
5. BPS	34.64 (8.20)	.52***	.49***	.50***	.56***

*Note. SBQ-R* Suicidal Behaviors Questionnaire-Revised, *PTSD* Posttraumatic Stress Disorder, *DSO* Disturbances in Self-Organization, *CPTSD* Complex posttraumatic stress disorder, *BPS* Borderline Pattern Scale.

*** p < .001.

### Parallel mediation analyses

#### Testing CPTSD and BP Symptoms as Mediators

**Fig. 1:**
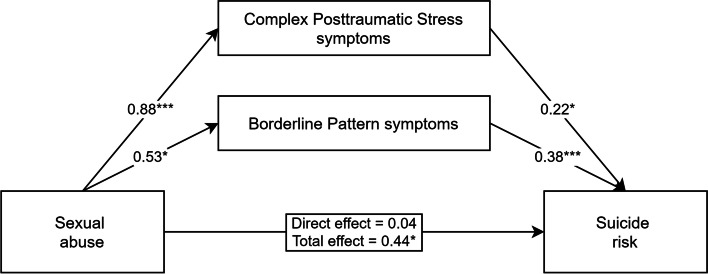
Parallel mediation model demonstrating the simultaneous effects of complex posttraumatic stress and borderline pattern symptoms on the relationship between sexual abuse and suicide risk severity. Adjusted for the covariates of age, gender, and timing of the traumatic experience (childhood vs. adulthood). Partially standardized coefficients for all paths from sexual abuse to mediators and outcome variable, and standardized coefficients for all paths from mediators to outcome variable are reported. * *p* < .05, ** *p* < .01, *** *p* < .001

Firstly, the total sum scores of CPTSD and borderline pattern symptoms were simultaneously included as mediators of the relationship between experiencing sexual trauma and SBQ-R scores. The direct and total effects, as well as the path coefficients for the parallel mediation analysis, are shown in Fig. 1. Over one-third (38.31%) of the total variance in suicide risk was accounted for by sexual abuse, both proposed mediators and covariates (F[6, 96] = 9.93, *p*<.001). The bootstrapping estimate revealed that indirect effects through the severity of complex posttraumatic stress (indirect = 0.19, 95% CI [0.008–0.440]) and borderline pattern symptoms (indirect = 0.20, 95% CI [0.054–0.395]) were statistically significant. Pairwise comparisons between the specific indirect effects revealed that they are not statistically different from each other (*β* = -0.01, 95% CI [-0.321–0.300]). When accounting for the effects of both mediators, paths, and covariates in the model, sexual trauma was no longer a significant predictor of suicide risk.

Regarding the covariates in the model, more severe suicide ideation was associated with younger age (*β* = -0.24, *p* = .004) and childhood trauma was associated with higher BP (*β* = 0.20, *p* = .048) symptom scores. The remaining relations were not statistically significant.

#### Testing PTSD, DSO, and BP Symptoms as Mediators

**Fig. 2:**
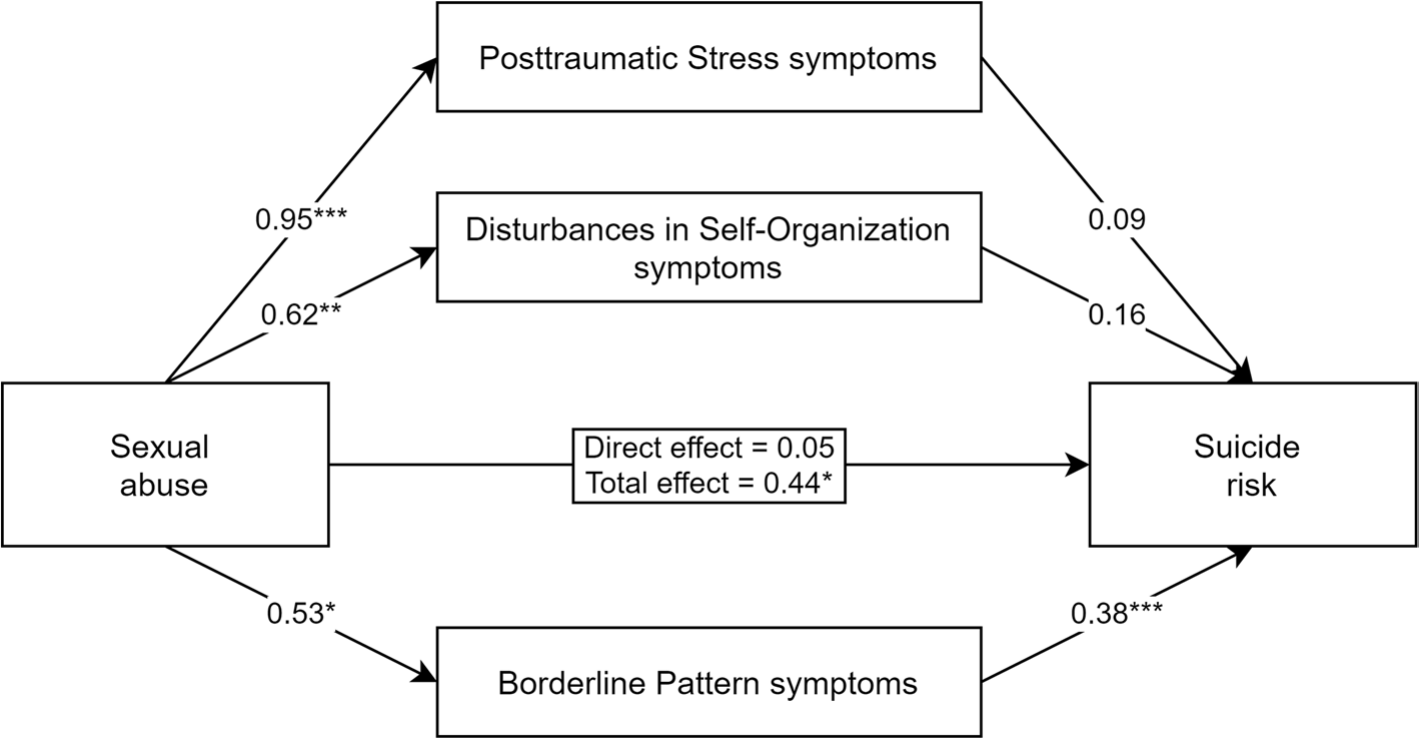
Parallel mediation model demonstrating the simultaneous effects of posttraumatic stress, disturbances in self-organization, and borderline pattern symptoms on the relationship between sexual abuse and suicide risk severity. Adjusted for the covariates of age, gender, and timing of the traumatic experience (childhood vs. adulthood). Partially standardized coefficients for all paths from sexual abuse to mediators and outcome variable, and standardized coefficients for all paths from mediators to outcome variable are reported. * *p* < .05, ** *p* < .01, *** *p* < .001

Next, we wanted to test the parallel mediation model with PTSD and DSO symptom scores included separately. This time the sum scores of PTSD, DSO, and BP symptoms were included as mediators (see Fig. 2). Similarly, as in the previous model, 38.36% of the total variance in suicide risk was accounted for by sexual abuse, proposed mediators, and covariates (F[7, 95] = 8.44, *p* <.001). In the current model, only BP symptoms were significantly associated with suicide risk. Relations with PTSD and DSO symptoms were no longer significant. The bootstrapping estimate revealed a significant indirect effect of sexual trauma, through the severity of borderline pattern symptoms, on suicide risk (indirect = 0.20, 95% CI [0.054–0.396]). Indirect effects through PTSD (indirect = 0.08, 95% CI [-0.134–0.301]) and DSO (indirect = 0.10, 95% CI [-0.046–0.280]) symptoms were not significant. When accounting for the effects of all three mediators, paths, and covariates in the model, sexual trauma was no longer a significant predictor of suicidal risk.

Regarding the covariates in this model, more severe PTSD symptom scores (*β* = −0.19, *p* = .036) and suicide risk (*β* = −0.25, *p* = .005) were associated with younger age. Childhood trauma was associated with higher DSO (*β* = 0.22, *p* = .028) and BP (*β* = 0.20, *p* = .048) symptom scores. The remaining associations were not statistically significant.

## Discussion

The main aim of the current study was to investigate the mediating role of complex posttraumatic stress disorder (CPTSD) and borderline pattern (BP) symptoms for the association between sexual abuse and suicide risk. In a parallel mediation model, both CPTSD and BP symptoms mediated the association between sexual trauma and suicide risk, following adjustment for the covariates of age, gender, and whether the traumatic experience occurred in childhood or adulthood. When PTSD and DSO symptoms were included in the model separately, only the borderline pattern symptoms remained a significant mediator. We also found that a large proportion, nearly 73%, of participants with CPTSD reported previous suicide attempt(s).

The results of the current study are in line with previous research demonstrating the associations between sexual trauma, suicide risk, and borderline personality disorder [10, 19, 20]. However, in this study, we found that not only a borderline personality pattern but also complex posttraumatic stress symptoms were significant mediators of the association between sexual abuse and suicide risk. Some previous studies showed that suicidal behaviors were more characteristic of BPD than CPTSD [7, 12]. However, our study reveals that for the victims of sexual abuse, the pathway to more severe suicide risk might also be formed through CPTSD symptoms. Our study also suggests that all complex PTSD symptoms are important for this mechanism. When PTSD and DSO symptoms were included in the model separately, the indirect effects were not significant anymore. This suggests that it is the combination of PTSD and DSO symptoms, rather than separate clusters of symptoms, which mediates the pathway from sexual trauma to suicide risk. In the current study, we also found very high rates of previous suicide attempts among participants with CPTSD. Other studies have shown that people with complex PTSD have a higher psychiatric burden than those with PTSD and those with no trauma-related diagnosis [28, 29]. We already have evidence that PTSD is a risk factor for suicide ideation, suicide attempts, and deaths by suicide [13–15], but more complex consequences of traumatic experiences might lead to an even greater suicide risk. Further research is needed to understand these associations better.

In the current study, we used a semi-structured clinician-administered interview for the assessment of the ICD-11 complex posttraumatic stress disorder symptoms. To our knowledge, this is the first study measuring the associations between sexual trauma, suicide risk, and complex posttraumatic stress with an in-depth interview constituted according to the ICD-11 CPTSD definition. Diagnostic interviews are based on the judgment of a trained clinician having knowledge of the assessed phenomenon, and are considered the gold standard for PTSD assessment [30]. To our knowledge, the ITI is the only clinician assessed measure for ICD-11 PTSD and CPTSD that has been evaluated psychometrically [22].

Some limitations of the study have to be taken into consideration. First of all, the sample size was relatively small. This could have affected the insignificant results of the second mediation model analysis via different PTSD and DSO symptom pathways. A larger sample could enable the analysis with more statistical power and generalizability. Furthermore, the sample was predominantly female. As a result, the findings are primarily applicable to women. Moreover, the sample was self-referred and not representative of the general population or clinical samples. Also, borderline pattern symptoms were evaluated using a self-report measure. Structured clinical interviews could ensure a more in-depth and accurate clinical assessment of BPD symptoms [30]. Furthermore, this was a cross-sectional study which is limited in determining causation. Longitudinal studies could enable more accurate causal analyses. Also, in the current study, we adjusted the analysis for the covariate of the timing of the sexual abuse (childhood vs. adulthood). However, it would be useful to separately evaluate the pathways from sexual abuse in childhood and later in life to suicide risk. For example, the results of the current analyses showed that childhood trauma was significantly associated with higher borderline pattern and disturbances in self-organization symptom scores. It might be important to additionally evaluate the role of other traumatic experiences in a person’s life, too. Therefore, conducting similar studies using in-depth assessment tools in different larger samples is highly recommended.

## Conclusions

This is the first study exploring the mechanisms of the pathways between sexual abuse and suicide risk through the symptoms of complex posttraumatic stress and borderline personality pattern, using a valid semi-structured tool for the clinical assessment of CPTSD. The results of the study suggest that when providing interventions for the victims of sexual abuse, suicide risk has to be assessed and managed for individuals with CPTSD as well as with BP symptoms. Although the results of the current study should be seen in the light of the aforementioned limitations, we hope it encourages future investigation of suicide risk and complex posttraumatic stress disorder among samples with sexual abuse and other traumatic experiences.

## Data Availability

The dataset of the current study is available from the corresponding author on reasonable request.

## Abbreviations

BP: Borderline pattern
CPTSD: Complex posttraumatic stress disorder
DSO: Disturbances in self-organization
ICD-11: International Classification of Diseases, 11^th^ revision
ITI: International Trauma Interview
PTSD: Posttraumatic stress disorder
